# Analysis of Social Performance and Action Units During Social Skills Training: Focus Group Study of Adults With Autism Spectrum Disorder and Schizophrenia

**DOI:** 10.2196/59261

**Published:** 2025-01-10

**Authors:** Hiroki Tanaka, Kana Miyamoto, Jennifer Hamet Bagnou, Elise Prigent, Céline Clavel, Jean-Claude Martin, Satoshi Nakamura

**Affiliations:** 1 International Christian University, Mitaka, Tokyo, Japan; 2 Nara Institute of Science and Technology, Ikoma, Japan; 3 Université Paris-Saclay, Orsay, France

**Keywords:** social performance rating scale, social skills training, autism spectrum disorder, schizophrenia, facial expressions, social, autism, training, communication, trainers, tool, neurological

## Abstract

**Background:**

Social communication is a crucial factor influencing human social life. Quantifying the degree of difficulty faced in social communication is necessary for understanding developmental and neurological disorders and for creating systems used in automatic symptom screening and assistive methods such as social skills training (SST). SST by a human trainer is a well-established method. Previous SST used a modified roleplay test to evaluate human social communication skills. However, there are no widely accepted evaluation criteria or social behavioral markers to quantify social performance during SST.

**Objective:**

This paper has 2 objectives. First, we propose applying the Social Performance Rating Scale (SPRS) to SST data to measure social communication skills. We constructed a Japanese version of the SPRS already developed in English and French. Second, we attempt to quantify action units during SST for people with autism spectrum disorder (ASD) or schizophrenia.

**Methods:**

We used videos of interactions between trainers, adults with ASD (n=16) or schizophrenia (n=15), and control participants (n=19) during SST sessions. Two raters applied the proposed scale to annotate the collected data. We investigated the differences between roleplay tasks and participant groups (ASD, schizophrenia, and control). Furthermore, the intensity of action units on the OpenFace toolkit was measured in terms of mean and SD during SST roleplaying.

**Results:**

We found significantly greater gaze scores in adults with ASD than in adults with schizophrenia. Differences were also found between the ratings of different tasks in the adults with schizophrenia and the control participants. Action units numbered AU06 and AU12 were significantly deactivated in people with schizophrenia compared with the control group. Moreover, AU02 was significantly activated in people with ASD compared with the other groups.

**Conclusions:**

The results suggest that the SPRS can be a useful tool for assessing social communication skills in different cultures and different pathologies when used with the modified roleplay test. Furthermore, facial expressions could provide effective social and behavioral markers to characterize psychometric properties. Possible future directions include using the SPRS for assessing social behavior during interaction with a digital agent.

## Introduction

Social communication skills are essential in human life. Experienced psychiatrists identify people with social communication difficulties, such as autism spectrum disorder (ASD or autism spectrum condition) and schizophrenia, through interviews based on diagnostic criteria, responses, and various neuropsychological tests [1,2]. To improve the identification accuracy of neurodevelopmental and neurological disorders such as ASD and schizophrenia, it is crucial to discover symptom-specific behavioral markers and phenotyping. Both adults with ASD and and adults with schizophrenia are characterized by social difficulties [3]; in terms of deficits, there have been reports describing conversational gestures that are less closely synchronized with co-occurring speech [4], frequency of eye contact [5], speech prosody [6], and delayed response time [7]. For schizophrenia, deficits in social cognition are a critical determinant of social functioning [8]. It has been reported that people with ASD and schizophrenia share several symptomatically similar characteristics such as flat or blunted affect (eg, reduced eye contact) and alogia (eg, impoverished speech) [9,10]. Thus, quantifying the degree of social communication difficulty is necessary to understand the nature of ASD and schizophrenia and to create systems for automatic symptom screening and early intervention methods such as social skills training (SST).

SST is a psychosocial treatment that has been widely applied and adapted to help people improve their social communication skills. It has been used in hospitals, employment support facilities, workplaces, and schools. A human trainer generally conducts SST to promote appropriate social communication skills, strengthen the individual’s social self-efficacy, and reduce social anxiety. The Bellack method [11], or step-by-step SST, is a well-structured approach that includes defining target skills, modeling, roleplay, feedback, and homework. This method defines the SST framework and its 4 basic skills: asking for requests, declining requests, telling positive feelings, and listening to others. Some researchers have been conducting studies to build SST systems using socially interactive agents [12,13]. Personalizing is an essential element for such SST systems. For example, Bellack et al [11] recommended using shorter and more precise feedback for people with schizophrenia who experience hallucinations. Since they often hear voices distracting the SST, focusing is difficult. For ASD, feedback could be disengaging if trainers frequently mention exaggerated gestures or facial expressions, since these are major symptomatic characteristics. Toward more personalized SST systems, it is imperative to quantitatively measure the characteristics of ASD and schizophrenia.

However, there are no widely accepted evaluation criteria during SST. If a well-validated scale was available, it could be used as a ground truth value for machine learning models in the SST system [14]. Among social behavioral markers, individual roleplay performance can be rated with a modified roleplay test conducted [15,16], although there is no internationally validated scale. This type of test is further limited because it must be evaluated by experts such as psychiatrists and SST trainers. Moreover, there is no robust social behavioral marker to quantify social performance during SST. One of the important behavioral markers is facial expression. Previous works showed that action units AU06 and AU12 are less expressed in ASD participants [17,18], while AU01 is also less expressed in ASD participants [19]. For patients with schizophrenia, as far as the authors know, no previous study has provided an analysis of particular action units, although machine learning–based schizophrenia detection models have been proposed [20].

This paper proposes applying the Social Performance Rating Scale (SPRS) to measure social communication skills. The SPRS is a validated scale developed in English [21] and French [22]. The English version was applied to videos of a simulated dinner party for participants with a primary diagnosis of social phobia, those with a primary diagnosis of anxiety disorder, and control participants. The French version was applied to videos of collaborative games between neurotypical participants. This scale has been extensively used in a variety of research [23]. In this paper, we newly developed a Japanese version of the SPRS that can be used without special qualifications in SST. We collected data on 4 SST tasks performed by adults with ASD, with schizophrenia, and control groups. Two raters used the SPRS to annotate their performance in SST. The goals of our study are (1) to validate our Japanese version of the SPRS, (2) to apply the SPRS to SST and examine the correlations between the SPRS and the modified roleplay test, (3) to investigate whether the SPRS can be applied to participants with ASD or schizophrenia, and (4) to analyze the relations between the SPRS, facial expressions, and questionnaire results. Here, we aim to analyze the differences in action units during SST roleplay in people with ASD or schizophrenia. This is an extended version of a conference workshop’s proceedings [24], in which we added an analysis of the facial expressions of people with ASD or schizophrenia.

In summary, we made contributions by developing and examining the following items: we developed a Japanese version of the SPRS; we analyzed social communication skills by focusing on the differences among adults with ASD or schizophrenia and a control group, on task differences of SST, and on questionnaire scores; and we analyzed action units by focusing on the differences among adults with ASD or schizophrenia and a control group, as well as questionnaire scores.

## Methods

### Ethical Considerations

This study was approved by the institutional review board of the Nara Medical University, Japan, and Nara Institute of Science and Technology, Japan (reference number: 2019-I-27‐2). We explained the experiment’s procedure and purpose to the participants and obtained informed consent. All data were anonymized. Participants received monetary rewards of up to 16,000 Japanese yen.

### Development of Japanese Version of the SPRS

The items in the English version of the SPRS are provided in Table 1, where each of them is rated on a Likert scale of 1 to 5 [25].

**Table 1.** Social Performance Rating Scale (SPRS; modified from an earlier study [21]).

Item	Description
Gaze	(1) Very poor: Participant completely avoids looking at the partner or stares continually. (5) Very good: Participant keeps eye contact during the conversation, does not stare; shifts focus during pauses and conversation.
Voice quality	(1) Very poor: (1) Participant speaks in a flat, monotonous voice; or (2) speaks at a low volume or mumbles; or (3) speaks overly loudly or has an intrusive tone (harsh or unpleasant voice quality). (5) Very good: Participant is warm and enthusiastic in verbal expression without sounding condescending or gushy.
Length	(1) Very poor: Monosyllabic (“hmmm,” “yeah,” “OK”) speech turns; or responses so long that the partner must interrupt or cannot utter a reply. (5) Very good: At most times, participant’s utterances are 2 or more sentences long. Participant acknowledges partner’s remarks without taking over and monopolizing the conversation.
Discomfort	(1) Very high: Complete rigidity of arms, legs, or whole body. Constant leg movements or fidgeting with hands, hair, or clothing. Extremely stiff face or constant facial tics. Frequent nervous throat clearing, swallowing, or stuttering. Frequent inappropriate giggling or laughing. Look of extreme discomfort and desire to flee the situation shown by 2 or more breaks in the role. Participant does not pay attention to the roleplay tasks most of the time. (5) Very low: Relaxed body posture and natural body movement. Participant laughs and smiles at appropriate times. She/he shows effective gesturing (to be distinguished from fidgeting). Participant focuses on the task all of the time, does not appear at all uncomfortable, but at ease in the situation.
Conversation flow	(1) Very poor: Participant makes few attempts to initiate the conversation. Even when prompted by the partner, participant cannot maintain the conversation. Participant uses almost no open-ended questions or is intrusive in questions and shows no empathy. Participant does not pay attention to the information provided by partner. (5) Very good: Participant easily maintains the conversation and responds smoothly to pauses in the conversation, often by following up on previous information provided by the partner or providing free information about themself on a related topic. Participant introduces new topics fluidly and frequently use open-ended questions. Participant shows genuine interest in the partner and follows up on the partner’s remarks with warmth or enthusiasm.

We developed a Japanese version of the SPRS. As suggested by an earlier work [26], in its guide to good practices in translation and cultural adaptation, we first translated the scale’s contents from English to Japanese and then from Japanese to English. This forward and backward translation method provides quality control to verify consistency between the translation and the original version. Inter Group Corp (Osaka, Japan), a company specializing in language and cross-cultural adaptation, translated the SPRS at our request. This Japanese version of the scale is available upon request to the authors.

### SST Roleplay

We used a Japanese SST dataset collected in a previous study [14]. We collected SST data at Nara Institute of Science and Technology for the control group and at Nara Medical University for the clinical group. The dataset includes data from 50 adults with the following characteristics: 16 in the ASD group, 15 in the schizophrenia group, and 19 in the control group. We collected these psychiatric or neurodevelopmental clinical groups to investigate their social communication difficulties. We included these groups because the main SST clients in clinical facilities are those with ASD and schizophrenia. The control group participants were hired by a recruitment agency. Two psychiatrists with SST experience joined this study as trainers (roleplay partners). All participants performed SST using roleplay and feedback from the trainer. Four basic skills were performed as follows: asking for requests, declining requests, telling about positive feelings, and listening to others (ie, asking, declining, telling, and listening). Each roleplay lasts 1 to 3 minutes. Some participants performed SST multiple times, although our analysis was done on the initial roleplay data.

We excluded from the control group participants who had undergone eye surgery or had a history of psychiatric hospitalization. For the clinical group, we excluded participants who scored less than 70 on the third edition of the Wechsler Adult Intelligence Scale [27]. We excluded participants with drug or alcohol addictions, personality disorders, or organic mental disorders. The data collection period was from January 2020 to January 2021. Due to COVID-19 concerns, a transparent partition was placed between the participants and trainers. Two video cameras were placed behind each conversationalist to record the other individual at chest level from the participants’ faces. We also recorded a video and used 2 Kinect (Microsoft Corporation) sensors installed at the side of each conversationalist.

### Questionnaire

We assessed each participant using the following questionnaires for correlation analysis with the SPRS and action units: the Facial Emotion Identification Test (FEIT) adopted in previous work [28], Kikuchi’s scale of social skills with 18 items (Kiss-18) [29], Singelis independent-interdependent Self-Construal Scale (Self-Construal Scale) [30], the second edition of the Social Responsiveness Scale (SRS-2) [31] (Japanese version [32]), and the Japanese version of the Brief Assessment of Cognition in Schizophrenia (BACS-J) [33]. In addition, we used the Autism Diagnostic Observation Schedule, Second Edition (ADOS-2), for the ASD group and the Positive and Negative Syndrome Scale (PANSS) for the schizophrenia group [2,34]. Where possible, we obtained the total score and subscales of each questionnaire. Excluding the FEIT, the questionnaires were collected before the SST data collection.

The FEIT, which assesses the emotional perception of facial emotions, includes facial images of 19 different people classified into 1 of 6 emotions: happiness, sadness, anger, surprise, fear, or disgust. We included this assessment because people with ASD and schizophrenia struggle with social cognition in facial images [34]. Kiss-18, which measures social skill levels, is comprised of 18 questions based on 6 social skill categories. This metric comprehensively measures social skills. Self-Construal Scale, which consists of questions on a 7-point rating scale, was developed to measure how people view themselves in relation to others. The SRS-2, composed of 65 questions, is an evaluation metric of the severity of social impairment. Although the SRS-2 was initially designed to assess people with ASD, it can also differentiate among various social communication difficulties. Its effectiveness has been investigated with clinical subjects and members of the general population [31].

To evaluate the participants during SST roleplay, we also adapted a modified roleplay test [15]. The scale is available only in Japanese. It includes items more closely related to psychopathology than the SPRS because the main target of this test is adults with schizophrenia. For this test, third-party psychiatrists watch recorded videos from both oblique and side views. Our 2 raters used a Likert scale from 1 to 5 for eye contact, body direction and distance, facial expression, voice variation, clarity, fluency, and social appropriateness. Since the required skills are situation-dependent, the social appropriateness differs depending on each SST task. Let us explain examples of social appropriateness for Bellack’s basic tasks. Listening to others, which determines whether the participants paid attention to the interlocutor, includes nodding, back-channels, and other nonverbal behaviors (eg, eye contact, smiling). In expressing positive feelings, social appropriateness involves expressing attention to the interlocutor’s responses and the suitability of the participant’s speech content. For making requests, social appropriateness assesses whether they explained the details of their request, including what kind of help they needed. It also includes whether they listened to the interlocutor. For declining, social appropriateness concerns whether they expressed remorse and appropriate reasons for their refusal. It also includes whether they proposed alternatives to the request (eg, “I’m sorry, but I propose doing it another way next time”), which is essential to this situation.

### SPRS Rating

Two third-party raters performed the annotations; all are nonexperts in psychiatry, psychology, or SST. The raters watched the recorded videos from oblique and side views for this evaluation. They initially evaluated all participants’ roleplay in the first of the 4 basic tasks during the SST. Here, they watched the first 3 videos and discussed the SPRS content for evaluation practice, and then they evaluated the scores of the other videos without discussion. They were not informed directly about the nature of the groups (clinical outpatients or control), but it is possible that they may have noticed the existence of these groups by the background in the video, since each group conducted SST in a different location. These raters were not given information about the other questionnaire scales, and they watched the videos in a shuffled order regardless of the group.

### Action Unit Extraction

The intensity of action units on OpenFace toolkit (version 2.0) [35] is measured in terms of mean and SD during individual roleplaying. From the action units extracted by OpenFace, we selected specific action units: AU01 (inner brow raiser), AU02 (outer brow raiser), AU04 (brow lower), AU06 (cheek raiser), and AU12 (lip corner puller). These action units represent various facial muscle activations. For our analysis, we referred to a previous study that focused on action units less directly associated with lip movement [36]. The intensity of each action unit ranges from 0 to 5, indicating a spectrum from no activation to high activation. First, the intensity of frames with less than 0.7 confidence in the data was excluded as not a number. We then applied a moving average filter on 3-second frames. We then calculated the mean and SD of the cleaned intensity data for each action unit to assess the intensity and variability, respectively.

### Statistical Analysis

The analyses were performed to investigate whether the SPRS could be used to evaluate social communication skills during SST. We analyzed the interrater agreement, items’ characteristics, construct validity, and internal consistency. To check the interrater agreement, we calculated the intraclass correlation coefficient (ICC, 2 k) [37]. We averaged the SPRS scores for the 2 raters for our analysis. We calculated the Spearman correlation coefficients to examine the homogeneity of the SPRS and the revised roleplay test. We assessed the factor structure of the SPRS by exploratory factor analysis with oblimin rotation to confirm the construct validity [38]. We examined the Bartlett test of sphericity (*P*<.001) and the Kaiser–Meyer–Olkin test for a sampling adequacy of 0.881 to apply the exploratory factorial analyses [39]. The internal consistency was assessed by calculating the Cronbach α coefficient [40] and McDonald ω [41].

We also analyzed the total of SPRS items in relation to the 3 groups, 4 tasks of SST, and the questionnaires. In comparing the control, ASD, and schizophrenia groups, we used the nonparametric Kruskal–Wallis test. Post-hoc analysis was conducted using the Mann-Whitney *U* test with Bonferroni correction. In comparing the asking, declining, telling, and listening tasks, we used the Wilcoxon signed rank test with Bonferroni correction. For action units, we applied the Mann-Whitney *U* test with Bonferroni correction to compare the control, ASD, and schizophrenia groups. Spearman correlation coefficients were calculated between the total SPRS items, action units, and questionnaire scores for all, control, ASD, and schizophrenia groups, although some questionnaires were not obtained from all participants. We used the JASP (Jeffreys’s Amazing Statistics program) for the statistical analysis [42].

## Results

### SPRS Statistics

We measured the interrater agreement by calculating the ICC (2 k) for validation of the SPRS annotated by the 2 raters. We opted for the random 2-way ICC method with the mean as the unit of evaluation, as shown in Table 2. The ICC ranged from 0 to 1, and all items had reliability values between 0.509 and 0.804. The ICCs were above 0.5, which indicates more than moderate reliability [22]. The score for each SPRS item was averaged for the 2 raters’ annotations in subsequent analyses. The Spearman correlation performed between the SPRS items and the modified roleplay test items shows significant results (all *P<.*001) and positive relationships. The correlation between all of them was greater than 0.6. Exploratory factor analyses were conducted on the 5 items of the SPRS using oblique rotation to investigate underlying constructs and assess dimensionality. Following a previous study [22], a 1-factor solution was adopted. Table 3 provides the factor loadings after rotation, illustrating how each of the 5 items aligns with the selected factor. The results show that the first factor explains 82.1% of the variance, indicating strong explanatory power. Factor loadings ranged from 0.874 for the “Discomfort” item to 0.932 for the “Conversation Flow” item, reflecting high values across all items. Additionally, Table 3 displays the uniqueness values for each variable, where uniqueness represents the proportion of variance specific to each variable that the factor does not explain. Higher uniqueness suggests lower relevance of that variable within the factorial model. For example, 23.6% of the variance in the “Discomfort” item remains unexplained by the factor, whereas the “Conversation Flow” item has a smaller unexplained variance (13.2%), showing greater alignment with the factor model. In sum, the factor analysis of the SPRS identifies a single factor composed of 5 items, capturing the verbal and nonverbal social skills essential for effective communication with others.

**Table 2.** Intraclass correlation coefficients for the Social Performance Rating Scale.

Item	Intraclass correlation coefficient
Gaze	0.509
Voice quality	0.795
Length	0.777
Discomfort	0.790
Conversation flow	0.804

**Table 3.** Factor loading for the Social Performance Rating Scale score.

Item	Factor 1	Uniqueness	Mean (SD)	Skewness	Kurtosis
Gaze	0.881	0.224	3.21 (1.00)	−0.351	−0.726
Voice quality	0.914	0.164	2.93 (1.18)	0.168	−0.975
Length	0.928	0.139	3.27 (1.10)	−0.247	−0.819
Discomfort	0.874	0.236	3.04 (1.02)	−0.074	−0.659
Conversation flow	0.932	0.132	3.15 (1.07)	−0.192	−0.585

We created an overall performance measure by summing the scores of the 5 SPRS items. This measure revealed that Cronbach α was 0.957 and McDonald ω was 0.958. These results indicate that the SPRS shows excellent internal consistency.

### SPRS Differences Among Participant Groups

**Figure 1.**
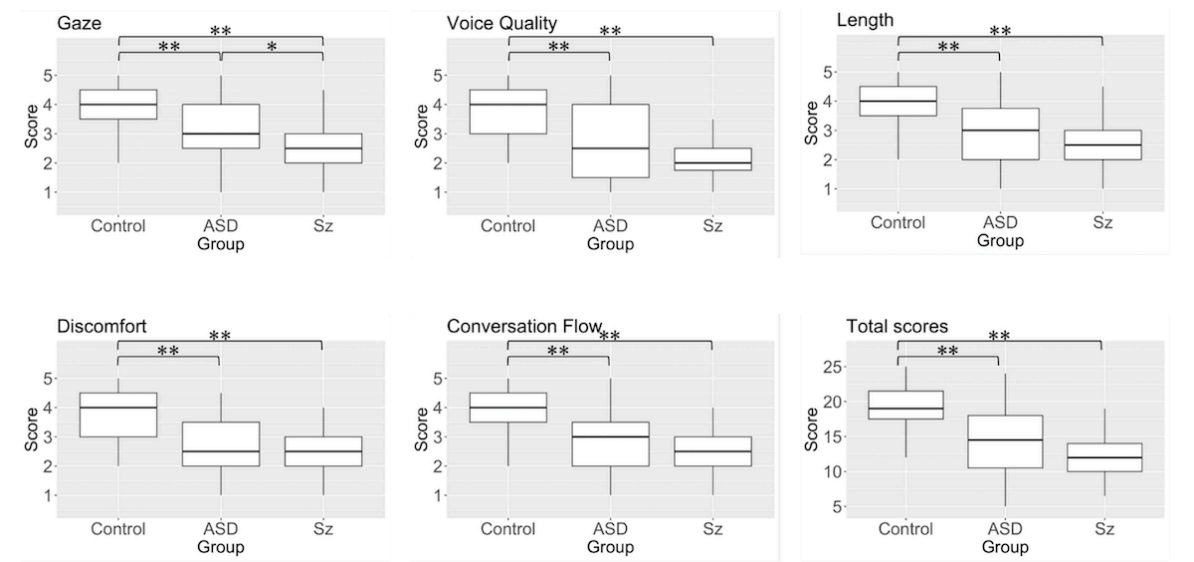
Group differences of Social Performance Rating Scale scores. ASD: autism spectrum disorder; Sz: schizophrenia. *P=.02, **P<.001.

Box plots of the 5 items of the SPRS and the total of the items for the control, ASD, and schizophrenia groups are shown in Figure 1. There were significant differences in all items and in the total of the SPRS items (*P<.*001). Post-hoc analysis was conducted using the Mann-Whitney *U* test with Bonferroni correction. We found significant differences in gaze, voice quality, length, discomfort, conversation flow, and the total of SPRS items for control-ASD and control-schizophrenia (*P<.*001). In ASD-schizophrenia, we found significantly greater gaze scores in the ASD group than in the schizophrenia group (*P*=.005).

### SPRS Differences Between 4 SST Tasks

SST task differences and a comparison are shown in Table 4. In the control group, we found significant differences in all items except gaze in asking-declining tasks and telling-declining tasks, and there were significant differences in the length of the asking-listening and telling-listening tasks. The declining task had lower scores than the asking and telling tasks for the control group. The length item of the listening task also had a low score compared with asking and telling tasks. We did not find significant differences between the 4 tasks in the ASD group. In the schizophrenia group, we found significant differences in the length of telling-listening tasks. For length, the listening task had a lower score than the telling task.

**Table 4.** Comparisons of Social Performance Rating Scale (SPRS) items for social skills training tasks. Parentheses represent significantly higher scored tasks. Effect size here is a rank-biserial correlation.

Item and combination			Control		Autism spectrum disorder		Schizophrenia	
			*P* value	Effect size	*P* value	Effect size	*P* value	Effect size
Gaze								
	Asking							
		Declining	.07	0.603	.08	−0.591	.58	−0.200
		Telling	.49	−0.289	.36	−0.295	.82	−0.091
		Listening	.48	0.219	.91	−0.044	.16	0.485
	Declining							
		Telling	.01	−0.743	.40	0.309	.97	0.026
		Listening	.15	−0.386	.17	0.491	.10	0.526
	Telling							
		Listening	.18	0.400	.24	0.500	.23	0.397
Voice quality								
	Asking							
		Declining	<.001 (Asking)^a^	0.963	.59	−0.250	.61	0.200
		Telling	.45	0.242	.45	−0.289	.22	−0.385
		Listening	.13	0.515	.81	0.091	.11	0.564
	Declining							
		Telling	.001 (Telling)^a^	−0.950	.97	−0.026	.12	−0.513
		Listening	.04	−0.600	.63	0.200	.49	0.255
	Telling							
		Listening	.28	0.341	.24	0.418	.03	0.758
Length								
	Asking							
		Declining	.001 (Asking)^a^	0.933	.52	0.198	.62	0.222
		Telling	.63	−0.167	.10	0.561	.43	−0.291
		Listening	.008 (Asking)^a^	0.824	.15	0.527	.04	0.727
	Declining							
		Telling	<.001 (Telling)^a^	−1.000	.30	0.364	.30	−0.364
		Listening	.56	−0.175	.24	0.350	.06	0.652
	Telling							
		Listening	.003 (Telling)^a^	0.912	.47	0.258	.005 (Telling)	0.923
Discomfort								
	Asking							
		Declining	.001 (Asking)^a^	0.933	.88	−0.073	.83	−0.111
		Telling	.90	−0.039	.81	0.111	.90	0.067
		Listening	.49	0.220	.60	−0.286	.29	0.382
	Declining							
		Telling	.001 (Telling)^a^	−0.912	.67	0.194	.67	0.238
		Listening	.02	−0.647	.62	−0.222	.25	0.385
	Telling							
		Listening	.41	0.248	.49	−0.267	.32	0.417
Conversation flow								
	Asking							
		Declining	.004 (Asking)^a^	0.797	.59	−0.179	.52	0.200
		Telling	.67	−0.164	.20	0.423	.15	−0.474
		Listening	.03	0.603	.50	0.242	.15	0.500
	Declining							
		Telling	<.001 (Telling)^a^	−0.949	.20	0.423	.15	−0.474
		Listening	.25	−0.342	.19	0.423	.37	0.318
	Telling							
		Listening	.02	0.700	>.99	0.010	.03	0.731
Total of SPRS items								
	Asking							
		Declining	<.001 (Asking)^a^	0.916	.41	−0.250	.96	0.036
		Telling	.63	−0.135	.61	0.162	.31	−0.330
		Listening	.07	0.510	.48	0.206	.03	0.681
	Declining							
		Telling	<.001 (Telling)^a^	−0.979	.38	0.276	.39	−0.267
		Listening	.09	−0.468	.27	0.333	.15	0.487
	Telling							
		Listening	.02	0.614	.53	0.192	.02	0.758

a Significant difference (*P<.*008).

### Action Units Differences Between Participant Groups

**Figure 2.**
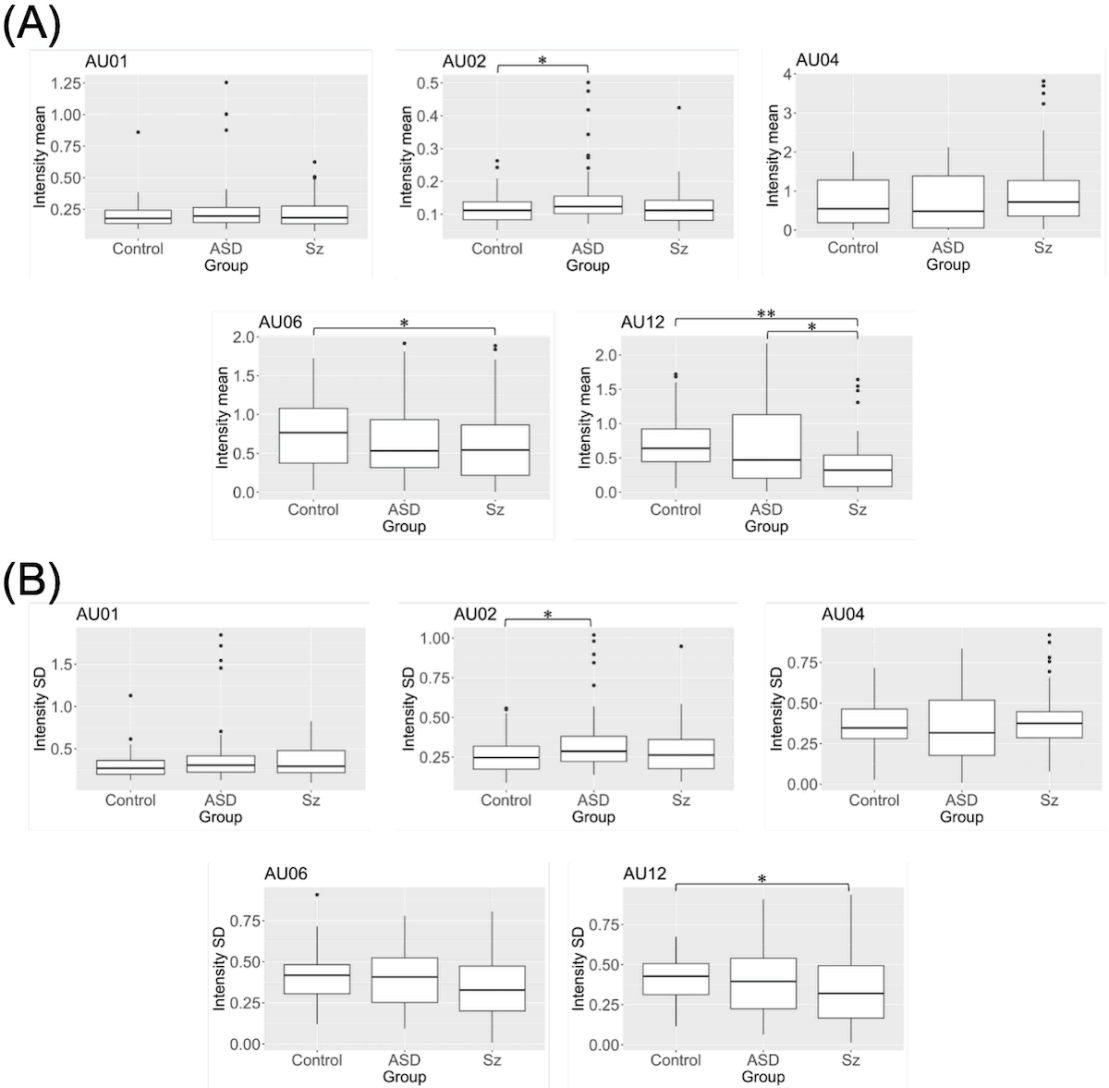
Action units’ intensities of mean values. (**A**) **P<.001, *P<.05 (AU02 control-ASD: *P*=.04, AU06 control-Sz *P*=.09, AU12 ASD-Sz *P*=.04); SDs. (**B**) *P<.05 (AU02 control-ASD: *P*=.02, AU12 control-ASD: *P*=.04). ASD: autism spectrum disorder; Sz: schizophrenia.

Participant group differences and comparisons, as well as mean and SD, are shown in Figure 2. We present the cases where significant differences were observed. We found that AU02 is significantly activated in people with schizophrenia compared with other groups. For mean values of the action units, this implies that AU02 is significantly less for the control group than the ASD group. For AU06, schizophrenia is significantly less than the control group. For AU12, it is more evident that schizophrenia is significantly less than either the control or ASD group. Regarding SD, people with ASD have significantly activated AU02 compared with the control group. For AU12, schizophrenia has significantly less variance than the ASD group.

### Correlation Between the SPRS, Action Units, and Questionnaire Scores

**Figure 3.**
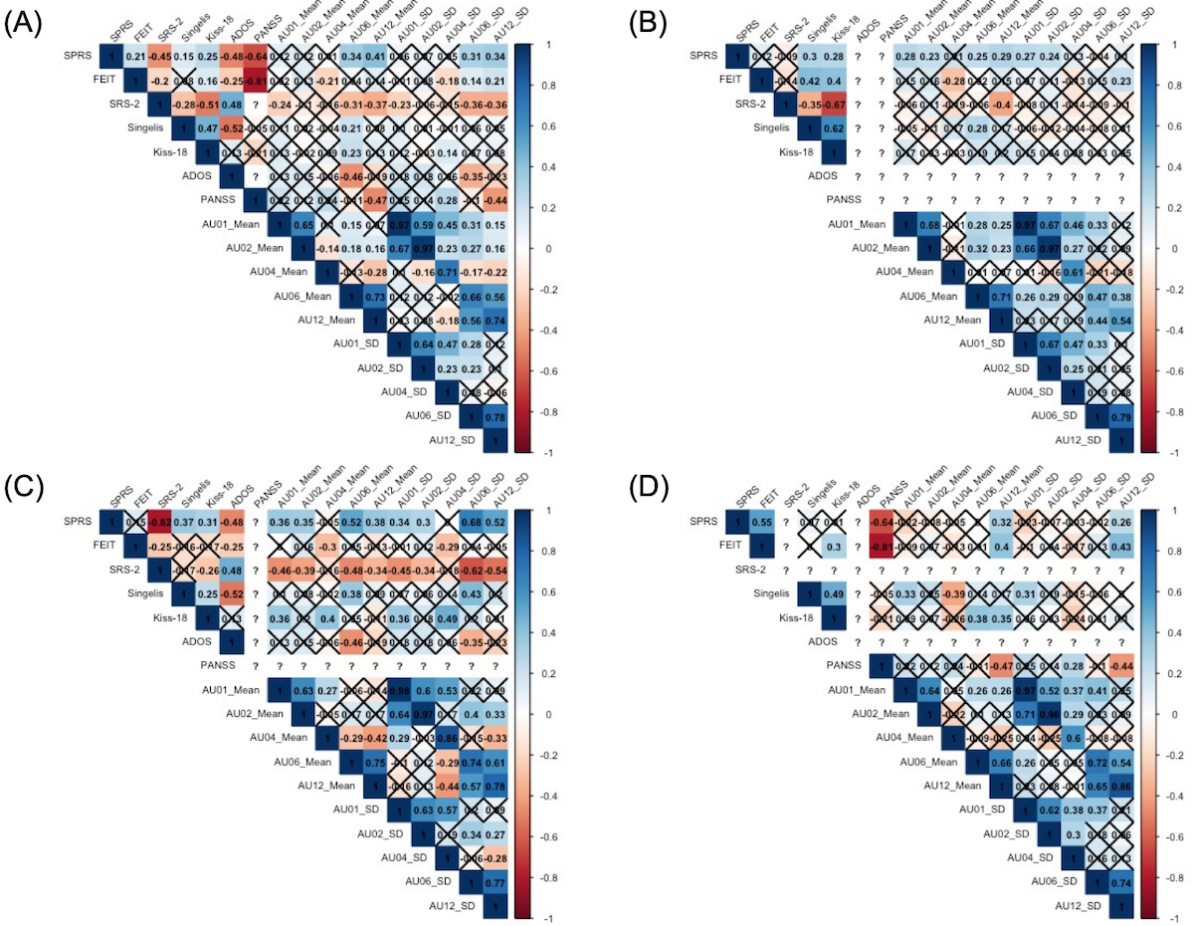
Correlation matrix (“?” indicates not applicable or missing data, cross mark represents nonsignificant correlations). (A) All groups, (B) control group, (C) ASD group, and (D) Sz group. ADOS: Autism Diagnostic Observation Schedule; FEIT: Facial Emotion Identification Test; Kiss-18; Kikuchi’s scale of social skills with 18 items; PANSS: Positive and Negative Syndrome Scale; SPRS: Social Performance Rating Scale; SRS-2: Social Responsiveness Scale.

Spearman correlation coefficients were calculated between the total SPRS items and questionnaire scores for all the following groups: control, ASD, and schizophrenia. The correlation coefficients are displayed in Figure 3. Across all groups, significant correlations were found between the total SPRS items and various measures, including the FEIT, SRS-2, Self-Construal Scale, Kiss-18, ADOS-2, PANSS, as well as the mean and SD values of AU06 and AU12. These findings suggest a strong relationship between social performance and various action unit statistics, as well as questionnaire scores.

In the control group, significant correlations with the total SPRS items were noted for Kiss-18 and the Self-Construal Scale, reflecting the cultural emphasis on harmony over assertiveness, which may influence social communication skills in Japan. In the ASD group, significant correlations were found with Kiss-18, the Self-Construal Scale, SRS-2, BACS-J, and ADOS-2, suggesting a relationship between these questionnaires and the social performance of individuals with ASD. Notably, significant negative correlations were observed in the SRS-2, BACS-J, and ADOS-2, highlighting the complexities of social interactions within this group.

In the schizophrenia group, significant correlations with the total SPRS items were found for the FEIT, BACS-J, and PANSS. These results suggest that emotional awareness and social performance are interconnected.

## Discussion

### Principal Findings

This is the first study applying the SPRS and analysis of action units for SST roleplay data. In this paper, we propose evaluating roleplay performance using the SPRS. We calculated the interrater reliability between 2 raters’ evaluated SPRS scores and found that the values were above 0.509 for any SPRS item. Our results show that the reliability was moderate to good [43]. Hamet Bagnou et al [22] calculated reliability among SPRS raters during collaborative tasks and showed that the reliability of gaze was the highest, but our results show that it has the lowest values. One reason could be the difference in conversation content. In our study, we applied 4 SST tasks, and the annotators needed to evaluate the SPRS based on the content of the tasks. The evaluation of SST tasks may have been more complex than that of collaborative tasks. Although there are slight differences between their study and ours, the results are nearly identical, with a reliability of 0.5 or higher in both studies.

The Spearman correlation between the SPRS items and revised roleplay test items was above 0.6. The factor analysis for the SPRS showed that the first factor explained 82.1% of the variance. The Cronbach α was 0.957, and the McDonald ω was 0.958. These results indicate that the SPRS shows excellent internal consistency and generally correlates to the modified roleplay test but is not identical.

We compared the SPRS scores by group and task. The control group differed significantly from the ASD and schizophrenia groups on every item of the SPRS. Several studies have shown that a control group shows higher social communication skills than an ASD group and a schizophrenia group, and we showed similar results.

The ASD group had a higher gaze score than the schizophrenia group. We consider it relevant that the eye movement abnormalities were severe in the schizophrenia group compared with the ASD group [44].

We conducted analyses of the differences in the SPRS scores between tasks for each group. In the control group, the declining task had lower scores than the asking and telling tasks, except for gaze. Declining is the most difficult task of the basic SST tasks, which may have contributed to the lower scores [11]. The listening task had lower scores than the asking and telling tasks in length. In the schizophrenia group, the listening task also scored lower than the telling task in length. The control group met the trainer for the first time in the data recording. It is possible that some control participants were not interested in the topics provided by the trainer in the listening task. The ASD and schizophrenia groups had met with the trainer several times. However, the schizophrenia group had low scores. Some participants with schizophrenia might have seemed disinterested in the conversation [11], and the raters might have annotated the low scores.

The control group showed that the SPRS had significant positive correlations with Kiss-18 and with the Self-Construal Scale.

The independence of the Self-Construal Scale measures is characterized by autonomy and distinction from others [45], and it is embedded within the culture [46]. In cultures with strong interdependence, harmony is an important concern, while assertiveness tends to be discouraged. This is predominant in the East, including Japan, and it may be related to social communication skills in Japan.

In the ASD group, there were significant positive or negative correlations between the SPRS and Kiss-18 or the Self-Construal Scale; this was also the case for the control group. We found significant negative correlations in the SRS-2, BACS-J, and ADOS-2. Our results suggest a relationship between the questionnaire for ASD and the social communication skills of the ASD group. The schizophrenia group had significant positive or negative correlations between the SPRS and the FEIT, BACS-J, and PANSS. Emotional awareness and communication skills are also related [47], and we surmise that our results show a significant difference in the FEIT. Another study showed that negative symptoms related to social motivation may be primary determinants of social outcomes in schizophrenia, potentially having a greater impact than social competence [48].

The results on action units also resemble the previous findings, in which an ASD group showed less smiling as represented in AU06 and AU12 [15,17], but there was no significant difference in terms of AU01 [18]. We found that AU02 was significantly activated in people with ASD compared with other groups. Moreover, this was the first study to conduct a detailed analysis of action units for schizophrenia. We also found that the AU06 and AU12 action units were significantly deactivated in people with schizophrenia compared with the control group. Accordingly, our results demonstrated that action units could work as social behavioral markers of people with ASD or schizophrenia.

### Limitations

This study is limited in terms of the population used and its generalizability. In particular, the SPRS was measured by 2 raters. Although we found high interrater correlations, a greater number of raters is needed to enhance the consistency and validity of the scale. In fact, previous work used an even larger number of raters, but a high interrater agreement was still maintained [21]. Our SST dataset was collected using outpatients with mild cases who were able to conduct SST verbally. Further study is needed to test whether the SPRS and action unit analysis could be applied to patients with more severe cases.

### Conclusions

We applied and validated a Japanese version of the SPRS to SST roleplay data to measure individual social communication skills. We analyzed the SST data of 4 tasks carried out by adults with ASD or schizophrenia as well as control participants. The results found significant differences between the control and ASD as well as schizophrenia in all SPRS items. We found significantly greater gaze scores in the ASD group than in the schizophrenia group. There were also task-based differences in the schizophrenia and control groups. Our results suggest that the SPRS can be a useful tool for assessing social communication skills in different cultures and different pathologies and, in addition, can evaluate SST effectiveness as well as the modified roleplay test. We also found that the action units numbered AU06 and AU12 were significantly deactivated in people with schizophrenia compared with the control group. Possible future directions include using the SPRS for assessing social behavior during interaction with a digital agent.

## Abbreviations

ADOS-2: Autism Diagnostic Observation Schedule, Second Edition
ASD: autism spectrum disorder
BACS-J: Brief Assessment of Cognition in Schizophrenia
FEIT: Facial Emotion Identification Test
ICC: intraclass correlation coefficient
JASP: Jeffreys’s Amazing Statistics Program
Kiss-18: Kikuchi’s scale of social skills with 18 items
PANSS: Positive and Negative Syndrome Scale
SPRS: Social Performance Rating Scale
SRS-2: Social Responsiveness Scale
SST: social skills training

## References

[1] Guha M (2014). Diagnostic and statistical manual of mental disorders: DSM-5 (5th edition). Ref Rev.

[2] Bastiaansen JA, Meffert H, Hein S (2011). Diagnosing autism spectrum disorders in adults: the use of Autism Diagnostic Observation Schedule (ADOS) module 4. J Autism Dev Disord.

[3] Marwaha S, Johnson S (2004). Schizophrenia and employment - a review. Soc Psychiatry Psychiatr Epidemiol.

[4] de Marchena A, Eigsti IM (2010). Conversational gestures in autism spectrum disorders: asynchrony but not decreased frequency. Autism Res.

[5] Senju A, Johnson MH (2009). Atypical eye contact in autism: models, mechanisms and development. Neurosci Biobehav Rev.

[6] Tanaka H, Sakti S, Neubig G, Toda T, Nakamura S Linguistic and acoustic features for automatic identification of autism spectrum disorders in children’s narrative.

[7] Heeman PA, Lunsford R, Selfridge E Autism and interactional aspects of dialogue.

[8] Dodell-Feder D, Tully LM, Hooker CI (2015). Social impairment in schizophrenia: new approaches for treating a persistent problem. Curr Opin Psychiatry.

[9] Konstantareas MM, Hewitt T (2001). Autistic disorder and schizophrenia: diagnostic overlaps. J Autism Dev Disord.

[10] Foss-Feig JH, McPartland JC, Anticevic A, Wolf J (2016). Re-conceptualizing ASD within a dimensional framework: positive, negative, and cognitive feature clusters. J Autism Dev Disord.

[11] Bellack AS, Mueser KT, Gingerich S (2013). Social Skills Training for Schizophrenia: A Step-by-Step Guide.

[12] Tanaka H, Iwasaka H, Matsuda Y, Okazaki K, Nakamura S (2021). Analyzing self-efficacy and summary feedback in automated social skills training. IEEE Open J Eng Med Biol.

[13] Nadel J, Grynszpan O, Martin JC (2022). The Handbook on Socially Interactive Agents: 20 Years of Research on Embodied Conversational Agents, Intelligent Virtual Agents, and Social Robotics Volume 2: Interactivity, Platforms, Application.

[14] Saga T, Tanaka H, Matsuda Y (2023). Automatic evaluation-feedback system for automated social skills training. Sci Rep.

[15] Sasaki T (2006). The reliability and validity of the revised role play test for assessment of social skills in schizophrenia. Clin Psychiatry.

[16] Tanaka H, Saga T, Iwauchi K (2023). The validation of automated social skills training in members of the general population over 4 weeks: comparative study. JMIR Form Res.

[17] Bangerter A, Chatterjee M, Manfredonia J (2020). Automated recognition of spontaneous facial expression in individuals with autism spectrum disorder: parsing response variability. Mol Autism.

[18] Alvari G, Furlanello C, Venuti P (2021). Is smiling the key? machine learning analytics detect subtle patterns in micro-expressions of infants with ASD. J Clin Med.

[19] Legiša J, Messinger DS, Kermol E, Marlier L (2013). Emotional responses to odors in children with high-functioning autism: autonomic arousal, facial behavior and self-report. J Autism Dev Disord.

[20] Bishay M, Palasek P, Priebe S, Patras I (2019). SchiNet: automatic estimation of symptoms of schizophrenia from facial behaviour analysis. IEEE Trans Affect Comput.

[21] Fydrich T, Chambless DL, Perry KJ, Buergener F, Beazley MB (1998). Behavioral assessment of social performance: a rating system for social phobia. Behav Res Ther.

[22] Hamet Bagnou J, Prigent E, Martin JC, Clavel C (2022). Adaptation and validation of two annotation scales for assessing social skills in a corpus of multimodal collaborative interactions. Front Psychol.

[23] Stevens S, Cooper R, Bantin T, Hermann C, Gerlach AL (2017). Feeling safe but appearing anxious: differential effects of alcohol on anxiety and social performance in individuals with social anxiety disorder. Behav Res Ther.

[24] Miyamoto K, Tanaka H, Bagnou JH Social performance rating during social skills training in adults with autism spectrum disorder and schizophrenia.

[25] Likert R (1932). A Technique for the Measurement of Attitudes Archives of Psychology de Marchena A.

[26] Wild D, Grove A, Martin M (2005). Principles of good practice for the translation and cultural adaptation process for patient-reported outcomes (PRO) measures: report of the ISPOR Task Force for Translation and Cultural Adaptation. Value Health.

[27] Tulsky DS, Chiaravalloti ND, Palmer BW (2003). Clinical Interpretation of the WAIS-III and WMS-III.

[28] Horan WP, Kern RS, Shokat-Fadai K, Sergi MJ, Wynn JK, Green MF (2009). Social cognitive skills training in schizophrenia: an initial efficacy study of stabilized outpatients. Schizophr Res.

[29] Kikuchi A (2007). Measure social skill: handbook of kiss-18 (110-115).

[30] Singelis TM, Sharkey WF (1995). Culture, self-construal, and embarrassability. J Cross Cult Psychol.

[31] Constantino JN, Gruber CP (2012). Social Responsiveness Scale: SRS-2.

[32] Kamio Y, Inada N, Moriwaki A (2013). Quantitative autistic traits ascertained in a national survey of 22 529 Japanese schoolchildren. Acta Psychiatr Scand.

[33] Kaneda Y, Sumiyoshi T, Keefe R, Ishimoto Y, Numata S, Ohmori T (2007). Brief assessment of cognition in schizophrenia: validation of the Japanese version. Psychiatry Clin Neurosci.

[34] Kay SR, Fiszbein A, Opler LA (1987). The positive and negative syndrome scale (PANSS) for schizophrenia. Schizophr Bull.

[35] Baltrusaitis T, Zadeh A, Lim YC, Morency LP OpenFace 2.0: facial behavior analysis toolkit.

[36] Yang L, Woo J, Achard C, Pelachaud C (2023). Exchanging... watch out!. arXiv.

[37] Shrout PE, Fleiss JL (1979). Intraclass correlations: uses in assessing rater reliability. Psychol Bull.

[38] Costello AB, Osborne J (2005). Best practices in exploratory factor analysis: four recommendations for getting the most from your analysis. Pract Assess Res Eval.

[39] Beavers AS, Lounsbury JW, Richards JK (2013). Practical considerations for using exploratory factor analysis in educational research. Pract Assess Res Eval.

[40] Cronbach LJ (1951). Coefficient alpha and the internal structure of tests. Psychometrika.

[41] McDonald RP (2013). Test Theory: A Unified Treatment.

[42] (2023). JASP (version 0.17). JASP.

[43] Koo TK, Li MY (2016). A guideline of selecting and reporting intraclass correlation coefficients for reliability research. J Chiropr Med.

[44] Shiino T, Miura K, Fujimoto M (2020). Comparison of eye movements in schizophrenia and autism spectrum disorder. Neuropsychopharmacol Rep.

[45] Kam C, Zhou X, Zhang X, Ho MY (2012). Examining the dimensionality of self-construals and individualistic–collectivistic values with random intercept item factor analysis. Pers Individ Dif.

[46] Markus HR, Kitayama S (1991). Culture and the self: implications for cognition, emotion, and motivation. Psychol Rev.

[47] Uzun G, Lok N (2022). Effect of emotional awareness skills training on emotional awareness and communication skills in patients with schizophrenia: a randomized controlled trial. Arch Psychiatr Nurs.

[48] Robertson BR, Prestia D, Twamley EW, Patterson TL, Bowie CR, Harvey PD (2014). Social competence versus negative symptoms as predictors of real world social functioning in schizophrenia. Schizophr Res.

